# Biomechanical study of the C5–C8 cervical extraforaminal ligaments

**DOI:** 10.1186/s13018-020-02006-9

**Published:** 2020-10-16

**Authors:** Qinghao Zhao, Yemei Yang, Penghuan Wu, Chengyan Huang, Rusen Zhang, Qingchu Li, Benchao Shi

**Affiliations:** 1 Department of Spinal Surgery, Zhujiang Hospital, Southern Medical University, 253 Gongye Middle Avenue, Guangzhou, 510282 China; 2 Academy of Orthopedics, Guangdong Province, Department of Orthopedics, The Third Affiliated Hospital of Southern Medical University, No. 183, Zhongshan Rd. West, Guangzhou, China; 3 Department of Dermatology, The Third Affiliated Hospital of Southern Medical University, No. 183, Zhongshan Rd. West, Guangzhou, China

**Keywords:** Cervical, Intervertebral foramina, Clinical anatomy, Extraforaminal ligaments, Biomechanical, Abduction injury

## Abstract

**Background:**

The anatomical distribution of the extraforaminal ligaments in the cervical intervertebral foramina has been well studied. However, detailed descriptions of the biomechanical characteristics of these ligaments are lacking.

**Methods:**

The paravertebral muscles were dissected, and the extraforaminal ligaments and nerve roots were identified. The C5 and C7 or C6 and C8 cervical nerve roots on both sides were randomly selected, and a window was opened on the vertebral lamina to expose the posterior spinal nerve root segments. Five needles were placed on the nerve root and the bone structure around the intervertebral foramen; the distal end of the nerve root was then tied with silk thread, and the weights were connected across the pulley. A weight load was gradually applied to the nerve root (50 g/time, 60 times in total). At the end of the experiment, segments of the extraforaminal ligaments were selectively cut off to compare the changes in nerve root displacement.

**Results:**

The displacement of the C5, C6, C7, and C8 nerve roots increases with an increasing traction load, and the rate of change of nerve root displacement in the intervertebral foramen is smaller than that in the nerve root on the outside area (*p* < 0.05). Extraforaminal ligaments can absorb part of the pulling load of the nerve root; the C5 nerve root has the largest load range.

**Conclusions:**

Cervical extraforaminal ligaments can disperse the tension load on the nerve root and play a role in protecting the nerve root. The protective effect of the C5 nerve root was the strongest, and this may anatomically explain why the C5 nerve roots are less prone to simple avulsion.

## Background

Daily activities of the human body cause the spinal nerves to be pulled [1]. Many different ligamentous structures in the spinal canal protect the nerve roots by preventing them from being pulled out from the spinal cord [2]. Grimes et al. [3] found that the lumbar extraforaminal ligaments (EFLs) significantly increased the ability of nerve roots to resist external traction and that this ability gradually increased from L3 to L5 in biomechanical experiments of axial stretching. Peretti et al. [4] described the fibrous tissue that pulls nerve roots toward the walls of the peripheral intervertebral foramina and considered these fibrous attachments to protect the nerve roots from traction-induced damage. However, all of these studies were conducted on lumbar EFLs, and biomechanical studies of EFLs in the cervical area are very rare.

Abduction injury of the upper limb often causes damage to the nerve roots or trunk of the brachial plexus. Brachial plexus injury is one of the most common peripheral nerve injury diseases, and also one of the disabling diseases. It not only has a huge physical and mental impact and burden on patients, but also brings a serious burden to the families and society of the patients. Kawai et al. [5] reported that the C5 nerves often have nerve trunk damage after the ganglia, while the C7 and C8 nerves often have nerve root damage before the ganglia, and damage to the C6 and T1 nerves is between these two groups in terms of severity. Most scholars believe that these differences are due to biomechanical differences among the nerve roots and the different anatomical features of each nerve root [5, 6]. How to accurately diagnose brachial plexus injury and provide individualized management is the research focus and direction of the surgeon. Therefore, a biomechanical study of the EFLs in the cervical region has important clinical value. We performed a detailed systematic anatomical description of the ligamentous structures in the inner, central and outer regions of the cervical intervertebral foramen; we believe that the ligamentous structures in the brachial plexus root region are stronger than those in the regions of other cervical segments [6].

In this study, biomechanical tests were performed on the ligaments of the brachial plexus nerve roots. The purpose of this study was to verify from a biomechanical point of view whether the EFLs in the cervical area exert a fixation effect on these nerve roots and whether there is difference in the fixation effect of the EFLs among different nerve roots. To explore the mechanism of different types of brachial plexus nerve root injury, we provide an objective biomechanical basis for the clinical diagnosis and treatment of related conditions.

## Materials and methods

**Fig. 1:**
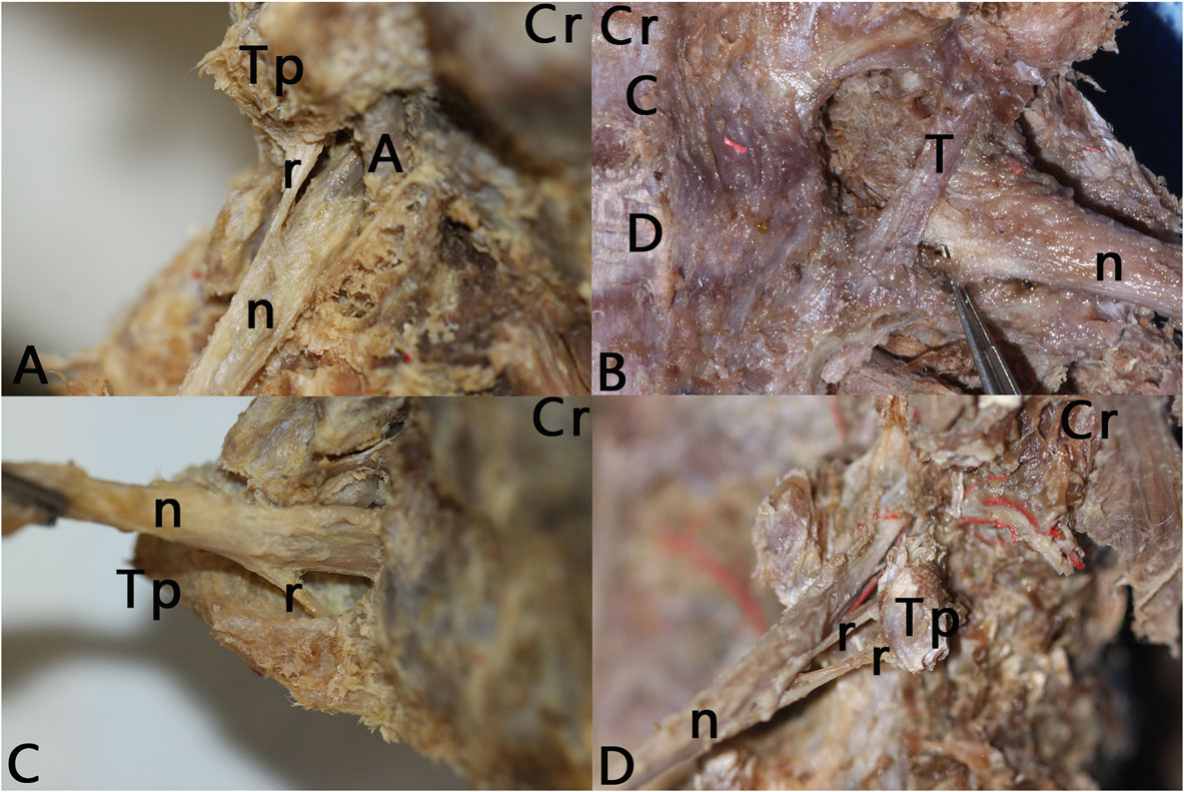
Ventral lateral views of intervertebral foramina. Cr, cranial; Tp, transverse process; A, vertebral artery; n, nerve root; C, cervical vertebral body; D, cervical intervertebral disc; T, transforaminal ligament; r, radiating ligament

Ten normal adult complete cervical spine formalin-embalmed specimens (7 males, 3 females, age 36–54 years, average age 43 years) without neck deformities, lesions or a surgical history were used for this study. The paravertebral muscles were routinely removed, and the integrity of the cervical nerve roots and the ligaments outside of the cervical intervertebral foramina was preserved (Fig 1). A unilateral opening was made in the window in the posterior cervical spine, as follows: the lamina, the yellow ligament and the medial facet of the corresponding facet joint were removed to form a square bone window approximately 1.5 cm in length, exposing the cervical nerve roots via a posterior approach. To preserve the integrity of the posterior cervical column as much as possible, the C5 and C7 nerve roots were exposed on one side of the specimen, and the C6 and C8 nerve roots were exposed on the other side. Anterior cervical plate and screw fixation was performed to limit vertebral motion. A denaturing powder was embedded in the lower end of the cervical spine specimen, and the specimen was fixed on an apparatus allowing three-dimensional motion of the spine.

**Fig. 2:**
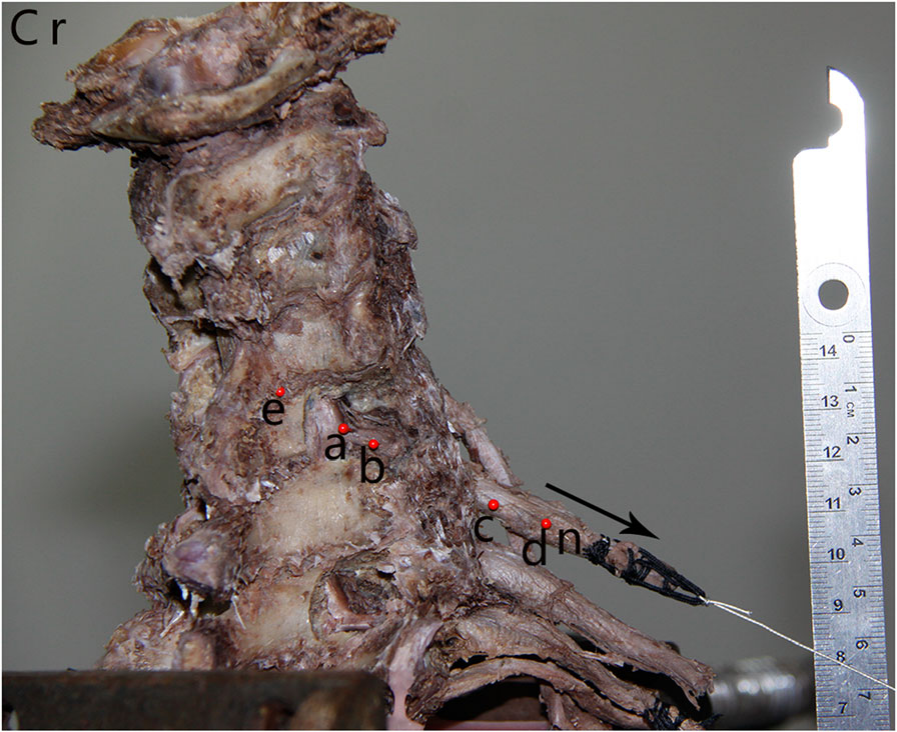
Cervical cadaver biomechanical model. Five positioning needles, named e, a, b, c, and d, placed along the axial direction of the nerve root. The arrow represents the pulling direction. Cr, cranial; La, lamina; n, nerve root; *, radiating ligament

The distal end of the nerve root was tied with silk thread, and a weight was connected to an attached pulley. The angle at which the nerve is pulled is based on the angle at which it emerges from the intervertebral foramen. Weights were used to gradually apply a load to the nerve root (50g/weight, 60 weights in total). A positioning needle (24 mm in length, 0.55 mm in diameter, and 2 mm in diameter at the red pearl head) was fixed to the vertebral lamina, and this point was denoted as point e. Two positioning needles were fixed to the nerve roots in the bone window, and these points were recorded as points a and b, respectively. Two other positioning needles were fixed to the nerve stem outside the intervertebral foramen, and these points were denoted as c and d, respectively. A straight line was made with the red pearl heads of the five positioning needles parallel to the axis of the nerve root. At this time, it should be noted that the intraspinal positioning needle was not inserted deeply, since the tip of the needle could enter other tissues after passing through the nerve root, which could seriously affect the experimental results. A scale (accurate to 1 mm) was placed next to the finished model for calibration during the subsequent measurements. The finished specimen model is shown in Fig. 2.

A Canon EOS 50D digital camera fixed on a tripod was used, and the camera position was adjusted such that the lens plane was parallel to the axis of the nerve root (f/5.6; 1/60 s; ISO: 200, focal length: 110 mm). After each loading, the camera recorded the position of each positioning needle head. Photoshop was used to measure the displacement of the positioning needles in the image.

**Fig. 3:**
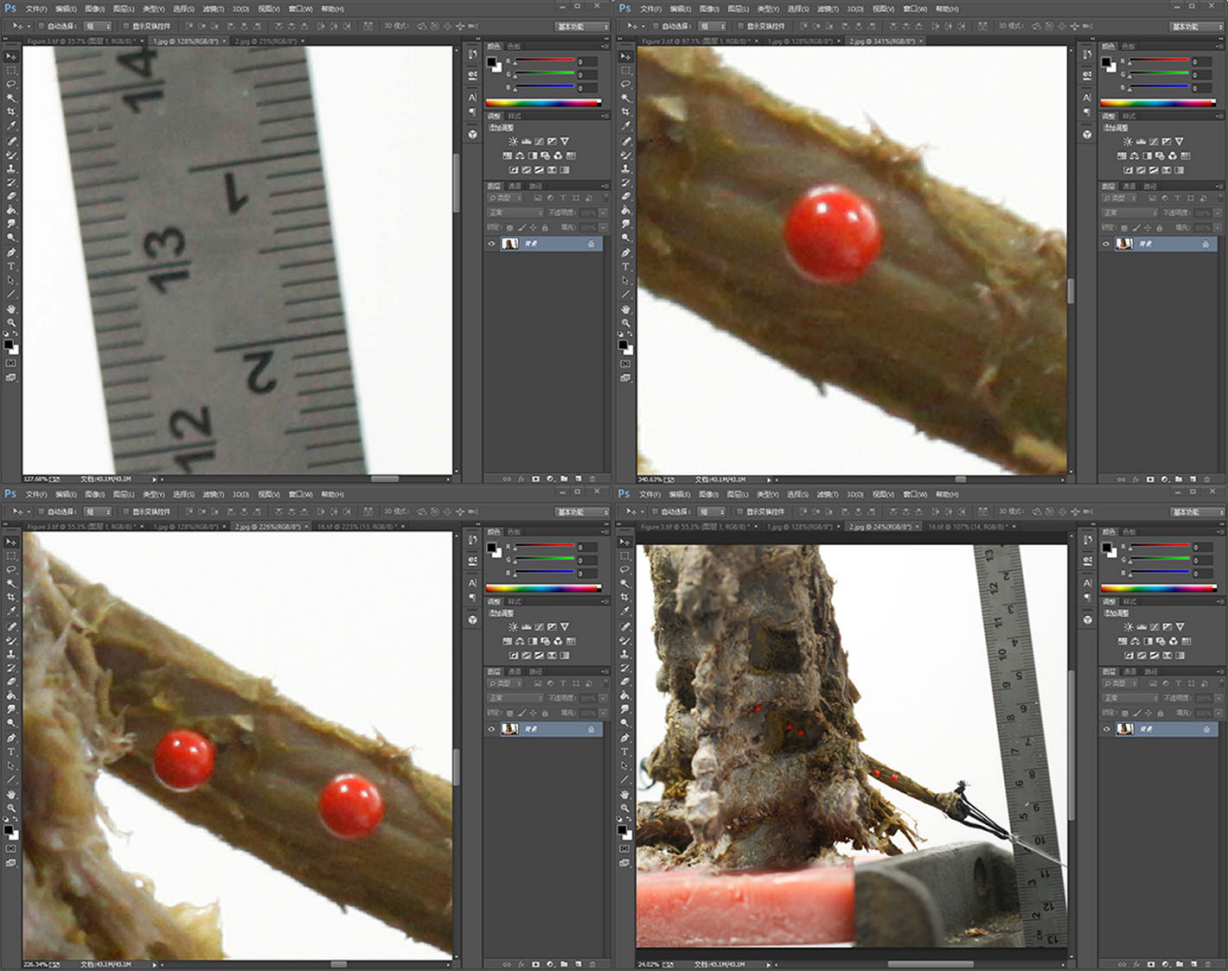
Displacement measurements determined using Adobe Photoshop software. **a** The measurement scale; *P*_1_, the intersection point of a marker line and the edge vertex of a scale line, with a length of 1 cm on the steel ruler; *P*_2_, the intersection point of a marker line and the edge vertex of a scale line, with a length of 2 cm on the steel ruler. **b** The center point; 0, the origin point; 1, the upper edge of the positioning needle; 2, the left edge of the needle on the left vertical ruler; 3, tangent to the lower edge of the needle; 4, the horizontal centerline; 5, the left vertical ruler tangent to the right edge of the needle; 6, the vertical centerline; Q, the center of the circular needle. **c** The measured distance between two points. **d** The overall view

The measurement scale was defined as follows: a mark line was drawn on the upper horizontal scale and placed on the scale line, with a length of 1 cm on the steel ruler. We ensured that the mark line intersected the edge vertex of the scale line (intersection point P1). Furthermore, another line was drawn and placed on the scale line, reaching 2 cm; we ensured that this line also intersected the vertex of the scale line (P2). Two lines were drawn from the left vertical ruler to intersect P1 and P2. After selecting the ruler tool on the left menu bar, P1 and P2 were connected, and the pixel count M between P1 and P2 was recorded. The number of millimeters per pixel was calculated as *W* = 10/M (mm) (Fig. 3a).

Once the center point was located, line 1 was drawn tangential to the upper edge of the positioning needle, and line 2 was drawn tangential to the left edge of the needle on the left vertical ruler. The intersection of lines 1 and 2 was defined as the origin *O*. Then, line 3 was drawn from the top horizontal ruler tangential to the lower edge of the needle, and the value *Y* was recorded in the information window. Then, line 4 was moved from the top of the horizontal ruler and dragged to a *Y* value of Y/2. Similarly, line 5 was drawn from the left vertical ruler tangential to the right edge of the needle, and the value of *X* was recorded in the information window. Then, line 6 was drawn from the left vertical ruler and dragged to an *X* value of X/2. The intersection of lines 4 and 6 was defined as the center “*Q*” of the circular needle (Fig. 3b). After defining these measurements, the distances between a and b, c and d, and e and b were measured and are labeled in the figure as Lab, Lcd, and Leb, respectively (Fig. 3c, d).

SPSS 20.0 statistical software was used for statistical processing of the measured data.

## Results

When axial loading was applied to the nerve root, it was obvious to the naked eye that the radiating ligaments outside the intervertebral foramina gradually tightened (Fig. 2) and that the needles c, d outside of the intervertebral foramina moved significantly, while the needles a, b inside of the spinal canal did not move significantly.

**Fig. 4:**
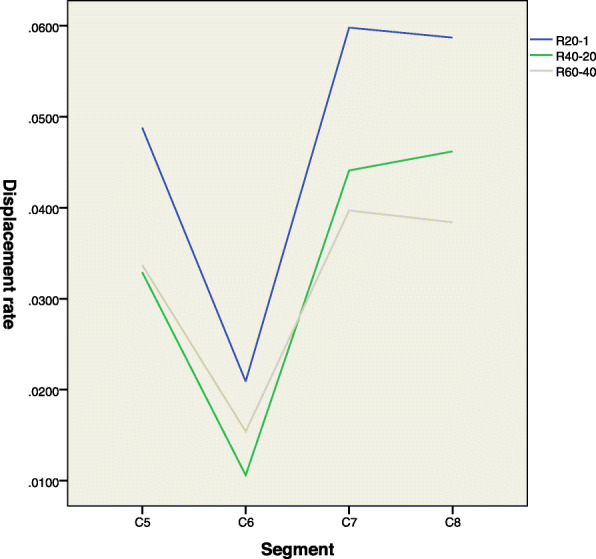
In this figure, R20-1 represents the rate of change in the displacement of the nerve root inside the spinal canal caused by increasing the load from 50 g (one weight) to 1000 g (20 weights). R40-20 represents the rate of change caused by increasing the load from 1000 g (20 weight) to 2000 g (40 weights). R60-40 represents the rate of change caused by increasing the load from 2000 g (40 weight) to 3000 g (60 weights). In the figure, we can see that the rate of change in the displacement of the C6 nerve roots inside the spinal canal is the smallest under each load, followed by those of the C5, C7, and C8 nerve roots, which were relatively high

When 50 g was gradually applied to the nerve root, the displacement of the inner e-b, a-b and outer c-d segments gradually increased. The displacement of segment e-b of the C5, C6, C7, and C8 nerve roots was plotted as a graph (Fig. 4). The rate of change in the displacement of the e-b segment (Reb) of the C5, C6, C7, and C8 nerve roots under loading was compared by ANOVA, as shown in Table 1, but no significant differences were observed (*p* > 0.05).

**Table 1:** The *p* values from a one-way ANOVA comparison of the variation rates in displacement of the eb segments of the C5, C6, C7, and C8 nerve roots “$\overline{\boldsymbol{x}}\pm \boldsymbol{s}$”

Nerve root	Variation rate		
	First stage	Second stage	Third stage
C5	0.06 ± 0.08	0.03 ± 0.04	0.05 ± 0.06
C6	0.02 ± 0.01	0.01 ± 0.02	0.02 ± 0.01
C7	0.06 ± 0.06	0.03 ± 0.03	0.05 ± 0.04
C8	0.05 ± 0.05	0.03 ± 0.03	0.04 ± 0.04
f	1.167	1.649	1.21
p	0.336	0.195	0.32

The first stage: the load increases from 50 g to 1000 g; The second stage: the load increases from 1000 g to 2000 g; the third stage: the load increases from 2000 g to 3000 g. *p* < 0.05 signifies that the difference is statistically significant; *f f*-statistic; *p p* value

Paired *t* tests were performed to compare the rate of change in the displacement of the a-b (Rab) and c-d (Rcd) segments of the C5, C6, C7, and C8 nerve roots. The results are shown in Table 2. By the table, we can see that the rate of change in the displacement of the a-b (Rab) and c-d (Rcd) segments of the C5, C6, C7, and C8 nerve roots in the first stage (the load increases from 50 g to 1000 g) and the second stage (the load increases from l000 g to 2000 g) had statistical significance, and only the rate of change in the displacement of the a-b (Rab) and c-d (Rcd) segment of the C5 nerve roots in the third stage (the load increases from 2000 g to 3000 g) had statistical significance, while the C6, C7, and C8 had no statistical significance. This result indicates that when the load within a certain range, the radiating ligaments absorb much of the tension that was applied to the nerve roots, making the displacement rate of the inner nerve root segment was significantly lower than that of the outer nerve root segment. The protective effect of the radiating ligaments on the C5 nerve roots is stronger for the tensile protection range of C5 nerve roots is wider.

**Table 2:** The results of the paired t tests comparing the Rab and Rcd of the C5, C6, C7, and C8 nerve roots

Segmental	First stage					Second stage			Third stage			
	Mean	sd	*t*	*p*	Mean	sd	*t*	*p*	Mean	Standard	*t*	*p*
C5	0.097	0.101	3.034	0.014	0.051	0.045	3.558	0.006	0.054	0.062	2.777	0.022
C6	0.082	0.089	2.900	0.018	0.050	0.038	4.129	0.003	0.019	0.071	0.851	0.417
C7	0.085	0.085	3.181	0.011	0.045	0.040	3.551	0.006	0.026	0.077	1.069	0.313
C8	0.068	0.054	3.958	0.003	0.032	0.029	3.491	0.007	0.029	0.053	-1.726	0.120

*sd* standard deviation; *p* < 0.05 represents a statistically significant difference; *t t*-statistic; *p p* value

**Fig. 5:**
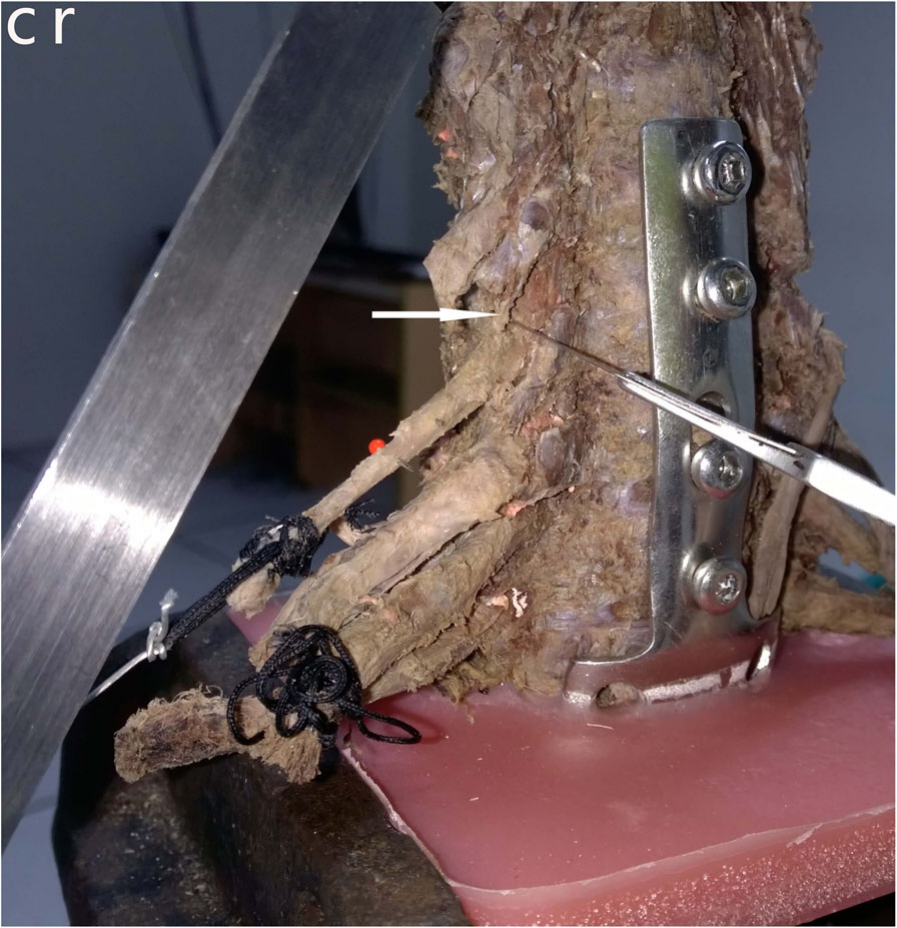
Removal of a superior radiating ligamentous structure (arrow) using a sharp knife. Cr, cranial

We found that when the radiating ligaments of the intervertebral foramen were cut, the displacement of the nerve root changed significantly, especially when the underlying radiating ligaments were cut; additionally, the change in the ligament displacement was the largest. When the external load reached 3000 g, the nerve root was often avulsed. However, when the transforaminal ligament was cut, the change in the nerve root displacement was negligible (Fig. 5).

## Discussion

At the end of the nineteenth century, there was much discussion regarding the protective mechanisms by which the intervertebral foramen could prevent nerve roots from being pulled out from the spinal cord [2, 7–13]. Kraan et al. [2] believed that the main mechanism was the action of the EFLs; when the spine was bent, the EFLs prevented the nerve roots from being compressed. However, Akdemir et al. [7] believed that the EFLs were not capable of reducing external axial pulling forces on nerve roots because they were too thin and easily broken.

We previously performed a detailed anatomical description of the ligamentous structures in the region of the cervical foramina [6, 14]. According to our observations, ligaments appear in both the inner and outer regions of the intervertebral foramen. The cervical EFLs are also relatively thin. They intersect the nerve roots almost vertically and connect the nerve roots to the surrounding intervertebral foraminal wall in a manner similar to the spokes of a bicycle wheel. According to this anatomical feature of the EFLs, they may serve to protect the nerve roots and retain them in the optimal position in the intervertebral foramen, providing both support and protection from friction with surrounding bony tissues. In addition, the EFLs can maintain the shape of the nerve root sleeve to a certain extent, which is conducive to proper electrophysiological nerve function and cerebrospinal fluid and blood circulation [15–17].

There are two types of EFLs in the cervical spine, namely, the radiating and transforaminal ligaments. The radiating ligaments act as cords to secure nerve roots to the vertebral transverse process, articular facet capsule, vertebral uncinate facet capsule, and pedicle [6]. Therefore, we believe that the radiating ligaments of intervertebral foramina serve as the primary mechanism protecting nerve roots against external traction.

Kraan et al. [2] studied the lumbar nerve roots of embalmed cadaveric specimens under mechanical loading and determined the displacement of the L1 to L4 nerve roots under traction at different sagittal angles for comparison. They found that the EFLs could reduce the longitudinal pulling force on the nerve roots. We found that the displacement rate of the nerve root segment in the spinal canal was lower than that of the nerve stem segment outside the intervertebral foramen under the same load. This finding suggests that the ligamentous structures of the intervertebral foramen play a role in protecting nerve roots against external traction. During the experiment, we found that the radiating ligaments of the cervical intervertebral area were pulled very tight and seemed to absorb much of the tension that was applied to the nerve roots. At the end of the experiment, the nerve root displacement increased significantly after the radiating ligaments were cut, while the displacement was not significantly changed after the transforaminal ligament was cut, indicating that the protective effect against traction was mainly due to the radiating ligaments, especially the underlying ligamentous radians.

In this experiment, we performed paired t-tests to individually analyze the displacement rate of the inner segment, in the spinal canal, and the outer segment, outside the intervertebral foramen, of the C5, C6, C7, and C8 nerve roots. We found that the displacement rate of the inner nerve root segment was significantly lower than that of the outer nerve root segment when the load was within 2000 g. Additionally, when the load was increased from 2000 g to 3000 g, only the displacement rate of the C5 nerve root segments showed a significant difference. This result indicates that in this load range, the EFLs still exert a protective effect on the C5 nerve roots, while the protective effect on the C6, C7, and C8 nerve roots is greatly weakened, which is consistent with the opinion of most clinical authors that the C5 nerve roots are not prone to simple root avulsion.

Formalin-embalmed cervical spine specimens were used in this study. The data may therefore be different from those in actual situations. The conclusion of this study is based on the findings of specimen-based experiments, so clinical verification is needed in the future.

## Conclusion

We believe that the radiating ligaments in the cervical foramina can transfer pulling forces on the nerve roots to the vertebral transverse process, articular facet capsule, vertebral uncinate facet capsule, and pedicle, among other structures, such dispersion of the load on the nerve roots would play a protective role. Ligaments exert the strongest protective effect on the C5 nerve roots, which may be an anatomical reason why the C5 nerve roots are not prone to simple root avulsion. A protective effect of the transforaminal ligament on the nerve roots was not obvious.

## Data Availability

The datasets used and/or analyzed during the current study are available from the corresponding author BS on reasonable request.

## Abbreviations

EFLs: Extraforaminal ligaments
